# Growth mixture modelling in families of the Framingham Heart Study

**DOI:** 10.1186/1753-6561-3-s7-s114

**Published:** 2009-12-15

**Authors:** Berit Kerner, Bengt O Muthén

**Affiliations:** 1 Department of Psychiatry and Biobehavioral Sciences, Semel Institute for Neuroscience and Human Behavior, University of California, 695 Charles E Young Drive South, Room 4357B, Box 951761, Los Angeles, California 90095-1761, USA; 2 Professor Emeritus, University of California, 2005 East Moore Hall, Los Angeles, California 90095, USA

## Abstract

Growth mixture modelling, a less explored method in genetic research, addresses unobserved heterogeneity in population samples. We applied this technique to longitudinal data of the Framingham Heart Study. We examined systolic blood pressure (BP) measures in 1060 males from 692 families and detected three subclasses, which varied significantly in their developmental trajectories over time. The first class consisted of 60 high-risk individuals with elevated BP early in life and a steep increase over time. The second group of 131 individuals displayed first normal BP, but showed a significant increase over time and reached high BP values late in their life time. The largest group of 869 individuals could be considered a normative group with normal BP on all exams. To identify genetic modulators for this phenotype, we tested 2,340 single-nucleotide polymorphisms on chromosome 8 for association with the class membership probabilities of our model. The probability of being in Class 1 was significantly associated with a very rare variant (rs1445404) present in only four individuals from four different families located in the coding region of the gene *EYA *(eyes absent homolog 1 in Drosophila) (*p *= 1.39 × 10^-13^). Mutations in *EYA *are known to cause brachio-oto-renal syndrome, as well as isolated renal malformations. Renal malformations could cause high BP early in life. This result awaits replication; however, it suggests that analyzing genetic data stratified for high-risk subgroups defined by a unique development over time could be useful for the detection of rare mutations in common multi-factorial diseases.

## Background

Longitudinal data analysis in genetic research is a new and emerging field with great potential. Genetic analysis of cross-sectional data generally assumes homogeneity in a sample with regard to the observed phenotype. However, longitudinal follow-up on the outcome variables often suggests that, nevertheless, individuals may differ in their development over time. These individual differences may even cluster into distinct subgroups with diverse environmental and genetic risk factors. A method that can be used to further explore this unobserved heterogeneity is growth mixture modelling (GMM) [1-4]. In GMM, the assumption of a single average growth curve is relaxed and different unobserved groups of individuals or latent subclasses are allowed to vary in their growth parameters, such as estimates of means, variances, and covariate influences. This flexible modelling framework allows for growth curves that differ in shape. Multi-level data, such as individuals nested in families, can easily be integrated into this model.

The Framingham Heart Study is one of the largest longitudinal clinical studies for which genetic material is available [5]. This study followed families over more than 50 years for common cardiac and metabolic disorders such as hypertension, diabetes, obesity, and heart disease. Current analyses have studied genetic and environmental risk factors in this sample by assuming a homogeneous population. In our study, we explore possible heterogeneity in this sample by relaxing the single-population assumption and allowing for parameter differences across unobserved subpopulations. Using GMM on systolic blood pressure (SBP) measures in 1060 males of the Original Cohort and the Offspring Cohort in 692 families, we detected three subclasses that varied significantly in their developmental trajectories. The growth curve of the first class (*n *= 60 individuals) was characterized by a high mean SBP early in life and an early, steep slope. The second class (*n *= 131 individuals) had a low mean SBP at a young age followed by a steep increase in SBP over time. The third class (*n *= 869 individuals) could be conceptualized as a normative class. Members of this subclass had low SBP at Exam 1 and the SBP remained low throughout the follow-up exams. Because previous studies had suggested a risk locus for high SBP in males on chromosome 8 in these data [6], we tested 2340 single-nucleotide polymorphisms (SNPs) on this chromosome for association with the class membership probabilities of our model. The probability of being in Class 1 was significantly associated with the coding SNP rs1445404 in the gene *EYA *(eyes absent homolog 1 in Drosophila) (*p *= 3.07 × 10^-13^). This very rare variant represents a miss-sense mutation in exon 3 of the gene. The minor allele of this SNP was present in only four individuals from four different families. Mutations in *EYA *were found in patients with brachio-oto-renal syndrome, as well as in individuals with isolated renal malformations [7,8]. Renal malformations can cause high blood pressure early in life. This result needs to be replicated, but it suggests that analyzing genetic data stratified for high-risk subgroups defined by a unique development over time could give an advantage for the detection of high-risk and rare mutations in common multi-factorial diseases.

## Methods

We used 1060 male individuals in 692 families from the Original Cohort and the Offspring Cohort of the Framingham Heart Study. SBP measured at four time points and spanning about 30 years of follow-up was used as outcome variable, whereas body mass index (BMI) and treatment for hypertension (HTNRX) were included in the model as time-varying covariates. Individuals with missing values on the covariates were excluded from the analysis, but missing values on the outcome variable at any time point were estimated with maximum-likelihood estimation under the assumption of missing at random (MAR) [9]. We fitted a three-level GMM by relaxing the assumption of identical parameter values across all mixture groups. The model was estimated by maximum likelihood using estimation-maximization (EM) algorithm. The first level described the variation over time, the second level described the variation over individuals, and the third level described the variation over families. Age was allowed to vary across the cohorts at each time point. We tested the fit of the model to the data by comparing the Bayesian information criteria (BIC) of the different class solutions for the non-nested models, with smaller BIC values indicating a better model fit [10]. The entropy of the classification and the posterior probability of belonging to a single class were taken into consideration as well. The analysis was performed with the computer software program Mplus [11].

The three-level growth mixture model for systolic blood pressure *y*_*tij *_for time point *t*, individual *i*, and pedigree *j *is described as follows using the individual-level latent class variable *c*_*ij *_with *K *classes. The Level 1 model is

where *e*_*tij *_follows a first-order autocorrelation structure. The Level 2 model is

where the *u *values are bivariate normal within latent class. The Level 3 model is

where the *v *values are bivariate normal within class and uncorrelated with the *u *values. The latent class probabilities follow a multinomial logistic regression with random intercepts,

where *ζ *is normally distributed.

The estimated class membership probability was then used as phenotype in a quantitative trait (QT) association analysis by testing one class against the other two classes performed with the software program GOLDENHELIX [12]. We tested 2340 SNPs on chromosome 8 for association with this phenotype. The significance of association was tested with the correlation/trend regression test under the basic, allelic model. A cut-off value of *p *< 10^-8 ^was used for genome-wide significance. We then performed 100,000 permutations of the data to evaluate the significance of our finding.

## Results

**Figure 1:**
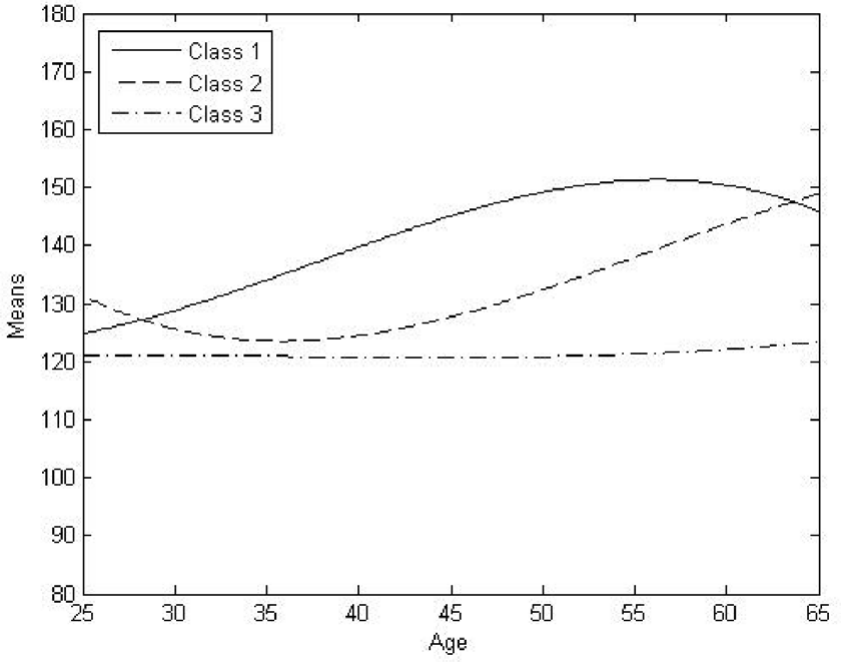
**Growth curves of the three latent classes over time**. Age is on the x-axis. The mean systolic blood pressure (SBP) is indicated on the y-axis.

We identified three distinct subgroups in this data set with regard to SBP development over time. The conventional 1-class random effect (multilevel) growth model was outperformed by a 2-class GMM in terms of BIC (BIC = 35527 for the 1-class model versus BIC = 33847 for the 2-class model). A 3-class model gave the lowest (best) BIC (BIC = 33845). In the 4-class model, BIC increased (BIC = 33865). The entropy for the 3-class model was not very high (entropy = 0.66). The classes varied in mean, slope, and shape of the growth curves (Figure 1). Class 1 consisted of 60 individuals with high SBP values early in life and a steep growth curve. Class 2 included 131 individuals. Members of this class started out with a low mean SBP, but developed a steep rise in SBP later in life. Class 3 contained a normative group of 869 individuals. The SBP measures in this group were normal and remained normal throughout follow-up.

Association analysis with SNPs on chromosome 8 revealed two signals with genome-wide significance. The first one was an association between Class 1 membership probability and SNP rs1445404 located in the third exon of the gene *EYA *(eyes absent homolog 1 in Drosophila) (*p *= 1.39 × 10^-13^, OR>8.1). A total of four individuals, one homozygote and three heterozygotes from four different families, had a rare C allele instead of the wild-type G allele (6.6% of the individuals in Class 1). This miss-sense mutation in exon 3 changes an alanine to a proline at amino acid position 20 of the protein, with likely consequences for the protein structure and folding of the protein. The homozygous individual and two of the heterozygous individuals belonged to Class 1. Interestingly, one heterozygous individual was assigned to Class 3; however, this individual was the youngest in this group of mutation carriers and the only one who was treated for hypertension as early as Exam 2. He also developed cardiac disease at age 49. This longitudinal course could indicate a misclassification due to very aggressive treatment of SBP at a very early stage.

The second signal was an association between Class 1 membership and the SNP rs6601495 located in the second exon of the gene *RP1L1 *(retinitis pigmentosa 1-like) (*p *= 3.8 × 10^-13^). This miss-sense mutation changes serine to threonine at amino acid position 112. Two individuals in this data set, one homozygote and one heterozygote, carried the rare C allele and both were members of Class 1. The homozygous individual was also homozygous for the SNP rs1445404. Whereas the C allele of this SNP is absent in the European and Asian population, homozygotes and heterozygotes for this variant are common among the Sub-Saharan African population (allele frequency of the C allele: 0.86). Whether this genomic variant indicates an ethnic admixture in this data set remains to be explored. Permutation testing with 100,000 permutations revealed a permutation *p*-value of 0.0001 for the single-marker permutation for marker rs1445404 and 0.0003 for marker rs6601495, indicating that of 100,000 permutations of the phenotype, only 10 and 30 permutations, respectively, reached the same or better results than indicated here. Permutation of the full model taking all the markers on chromosome 8 into account revealed a permutation *p*-value = 0.0044 for marker rs1445404, and 0.0085 for marker rs6601495 after Bonferroni correction for multiple testing.

## Discussion

We demonstrated here that GMM is a powerful tool to address unobserved population substructure in longitudinal data sets. By assigning individuals into different risk groups based on phenotype development over time, we were able to identify rare genomic variants that were present only in one group and absent in the others. Our approach used a two-stage design, in which we first defined class membership probabilities, and in a second step performed a quantitative trait association analysis. This approach may be biased. The low number of individuals who carried the identified rare mutations prohibited incorporating the genotype information of the SNPs directly into the model. The fact that carriers of these rare mutations were found only in one class and not in the other classes, however, justified our approach. Our study was limited by the very small size of the high-risk group, which might lead to spurious associations. In order to correctly interpret our finding, it would be necessary to replicate the results in a larger sample. Because statistical replication might require very large data sets, given the very rare nature of the mutation, an alternative approach would be a biological validation. Functional consequences of the mutation could be tested for by renal ultrasound or renal function tests in the affected individuals. A further limitation of our approach is the sensitivity to population stratification and admixture.

## Conclusion

GMM is a useful tool to detect subgroups in heterogeneous populations. We demonstrate here that family structure can easily be incorporated into the model. We successfully identified a high-risk group with steep growth over time. Members of this latent class had high blood pressure early in life with continuous steep increase. The class membership probability showed significant association with a rare mendelian variant in a gene that is involved in renal development. However, for correct interpretation of this result, replication in a larger sample or biological validation would be essential. Our approach may be a useful complement to the commonly used case/control association design because it provides more power to identify rare variants associated with a severe phenotype.

## List of abbreviations used

BIC: Bayesian information criterion; BMI: Body mass index; EM: Estimation maximization; GMM: Growth mixture model; HTNRX: Treatment for hypertension; QT: Quantitative trait; MAR: Missing at random; SBP: Systolic blood pressure; SNP: Single-nucleotide polymorphism.

## Competing interests

Dr. B. Kerner declares to have no financial interest or potential conflicts of interest. Dr. B. Muthen is a co-developer of the commercial computer program Mplus used in the analyses.

## Authors' contributions

BK carried out some of the growth mixture modelling and the association testing of the SNPs. She drafted the manuscript. BOM designed the growth mixture model and performed some of the analyses, wrote the input for Mplus, supervised the modelling, and edited the paper. BK and BOM participated equally in the design of the study and the interpretation of the results. Both authors read and approved the final manuscript.
